# Effects of Menthol on Nicotine Pharmacokinetic, Pharmacology and Dependence in Mice

**DOI:** 10.1371/journal.pone.0137070

**Published:** 2015-09-10

**Authors:** Shakir D. Alsharari, Justin R. King, Jacob C. Nordman, Pretal P. Muldoon, Asti Jackson, Andy Z. X. Zhu, Rachel F. Tyndale, Nadine Kabbani, M. Imad. Damaj

**Affiliations:** 1 Department of Pharmacology and Toxicology, College of Pharmacy, King Saud University, Riyadh, Kingdom of Saudi Arabia; 2 Department of Pharmacology and Toxicology, Medical College of Virginia, Virginia Commonwealth University, Richmond, Virginia, United States of America; 3 Department of Molecular Neuroscience, Krasnow Institute for Advanced Study, George Mason University, Fairfax, Virginia, United States of America; 4 Campbell Family Mental Health Research Institute, Centre for Addiction and Mental Health (CAMH), Department of Pharmacology and Toxicology, and Psychiatry, University of Toronto, Toronto, Ontario, Canada; Case Western Reserve University School of Dental Medicine, UNITED STATES

## Abstract

Although menthol, a common flavoring additive to cigarettes, has been found to impact the addictive properties of nicotine cigarettes in smokers little is known about its pharmacological and molecular actions in the brain. Studies were undertaken to examine whether the systemic administration of menthol would modulate nicotine pharmacokinetics, acute pharmacological effects (antinociception and hypothermia) and withdrawal in male ICR mice. In addition, we examined changes in the brain levels of nicotinic receptors of rodents exposed to nicotine and menthol. Administration of i.p. menthol significantly decreased nicotine’s clearance (2-fold decrease) and increased its AUC compared to i.p. vehicle treatment. In addition, menthol pretreatment prolonged the duration of nicotine-induced antinociception and hypothermia (2.5 mg/kg, s.c.) for periods up to 180 min post-nicotine administration. Repeated administration of menthol with nicotine increased the intensity of mecamylamine-precipitated withdrawal signs in mice exposed chronically to nicotine. The potentiation of withdrawal intensity by menthol was accompanied by a significant increase in nicotine plasma levels in these mice. Western blot analyses of **α**4 and **β**2 nAChR subunit expression suggests that chronic menthol impacts the levels and distribution of these nicotinic subunits in various brain regions. In particular, co-administration of menthol and nicotine appears to promote significant increase in **β**2 and **α**4 nAChR subunit expression in the hippocampus, prefrontal cortex and striatum of mice. Surprisingly, chronic injections of menthol alone to mice caused an upregulation of **β**2 and **α**4 nAChR subunit levels in these brain regions. Because the addition of menthol to tobacco products has been suggested to augment their addictive potential, the current findings reveal several new pharmacological molecular adaptations that may contribute to its unique addictive profile.

## Introduction

Menthol is a commonly used additive in tobacco products. In cigarettes, menthol has been shown to affect a smoker's exposure to nicotine [1], [2] and smokers who use mentholated cigarettes have lower cessation rates in standardized treatment programs than smokers who use non-menthol cigarettes [3], [4], [5]. Although many factors have been implicated in the initiation and dependence of menthol cigarettes, earlier studies showed that menthol itself inhibits nicotine metabolism [1] and that menthol cigarette smoking leads to elevated serum nicotine and cotinine levels and greater exhaled carbon monoxide (CO) levels [2]. These elevated levels of nicotine may influence smoking dependence and intake since nicotine mediates most of the pharmacological and addictive properties of tobacco. Nicotine administration is known to result in several physiological and pharmacological effects and to produce subjective feelings of reward and pleasure in humans and in animal models. Furthermore, the interaction between menthol and nicotinic acetylcholine receptors (nAChRs) has been examined previously in both *in vivo* and *in vitro* studies [6], [7], [8], [9]. For example, irritation and sensory perception induced by nicotine [6] and cigarette smoke inhalation [8] are significantly reduced by menthol. Menthol's ability to trigger the cold-sensitive transient receptor potential melastatin (TRPM) receptor is thought to be a mechanism for the cooling sensation it provokes when inhaled, eaten, or applied to the skin. In addition to sensory responses, nicotine-induced decreases in body temperature, due to cutaneous vasodilation, are significantly diminished after both chronic and acute menthol administrations [7].

In the central nervous system, nicotine binds selectively to a class of nAChRs which can be broadly divided into two subgroups: heteromeric **β**-subunit containing and homomeric **α**7 receptors [10], [11]. Recently menthol has been shown to regulate the function [9] and levels [12] of α4β2* (* means additional subunits are possible) nAChRs in the brain and a direct effect on nAChRs in cultured cells. To date however, little is known about menthol’s actions on other nAChRs subtypes in the brain and periphery. In this study we investigated the effects of menthol on nicotine examining both the altered pharmacokinetics and the resulting effects on nicotine pharmacodynamics and withdrawal in the mouse. In addition, we determined if menthol administration in the mouse affects the extent of **α4β2** nAChR upregulation in the brain after chronic exposure to nicotine in the mouse. We hypothesized that inhibition of nicotine metabolism by menthol should both increase nicotine’s plasma levels and decrease nicotine’s systemic clearance substantially enhancing nicotine pharmacological effects and **α4β2** nAChRs upregulation.

## Materials and Methods

### Animals

Male adult ICR mice (20-25g) obtained from Harlan Laboratories (Indianapolis, IN) were used throughout the study. Animals were housed in an AALAC approved facility in groups of five and had free access to food and water. Experiments were performed during the light cycle and were approved by the Institutional Animal Care and Use Committee of Virginia Commonwealth University.

### Drugs

(−)-Nicotine hydrogen tartrate salt [(−)-1-Methyl-2-(3-pyridyl) pyrrolidine (+)-bitartrate salt], (-)-menthol and mecamylamine hydrochloride were purchased from Sigma-Aldrich (St. Louis, MO). All drugs except for menthol were dissolved in physiological saline (0.9% sodium chloride) and injected at a total volume of 1ml/100 g body weight unless noted otherwise. Menthol was dissolved in a mixture of 1:1:18 [1 volume ethanol/1 volume Emulphor-620 (Rhone-Poulenc, Inc., Princeton, NJ) and 18 volumes distilled water] and administered intraperitoneal (i.p.). All doses are expressed as the free base of the drug. Mecamylamine and nicotine were injected subcutaneously (s.c.).

### Behavioral Tests

#### Tail-flick test

The antinociceptive effect of drugs was assessed by the tail-flick assay. A control response (2- to 4-s latency) was determined for each mouse before treatment, and test latency was determined after drug administration. To minimize tissue damage, a maximum latency of 10 s was imposed. Groups of 8 to 12 mice were used for each dose and treatment condition. Antinociceptive response was calculated as percentage of maximum possible effect (%MPE), where %MPE = [(test value − control value)/(cutoff (10 s) − control value)] × 100.

*Hot-plate Test*. Mice were placed into a 10-cm wide glass cylinder on a hot plate (Thermojust Apparatus, Columbus, OH) as a measure of antinociception. The hot plate was a rectangular heated surface surrounded by Plexiglas and maintained at 55°C. The device was connected to a manually operated timer that recorded the amount of time the mouse spent on the heated surface before showing signs of nociception (e.g., jumping, paw licks). Two control latencies at least 10 min apart were determined for each mouse. The normal latency (reaction time) of 8 to 12 s was assessed with a saline injection. To avoid tissue damage, the hot plate automatically disengaged after 40 s. Groups of 8 to 12 mice were used for each dose and treatment condition. Antinociceptive response was calculated as %MPE, where %MPE = [(test value − control)/(cutoff time (40 s) − control) × 100]. The reaction time was scored when the animal jumped or licked its paws.

#### Body temperature

Rectal temperature was measured by a thermistor probe (inserted 24 mm) and digital thermometer (Yellow Springs Instrument Co., Yellow Springs, OH). The difference in rectal temperature before and after treatment was calculated for each mouse. The ambient temperature of the laboratory varied from 21–24°C from day to day.

*Somatic Signs*. Mice were observed for 20 min in individual clear Plexiglas cages for typical somatic withdrawal behaviors and any unique behavior. Mice were individually housed overnight prior to the test day. Typical withdrawal signs that were tallied included paw tremors, writhing, scratching, backing, body tremor and head shakes. The total number of signs displayed by mice during the 20 min observation period were counted. Somatic signs were calculated as the mean ± S.E.M. All observations were done in a blinded manner.

#### Elevated plus-maze

An elevated plus-maze, prepared with gray Plexiglas, consisted of two open arms (23 × 6.0 cm) and two enclosed arms (23 × 6 × 15 cm in wall height) that extended from a central platform (5.5 × 5.5 cm). The maze was mounted on a base raised 60 cm above the floor. Fluorescent lights (350 lux intensity) located in the ceiling of the room provided the only source of light to the apparatus. The animals were placed in the center of the maze, and the time spent in the open arms was automatically recorded by a photocell beams system. The number of arm crosses in the plus maze test was also counted as a measure of locomotor activity. The test lasted 5 min, and the apparatus was thoroughly cleaned after removal of each animal. Results were expressed as percentage of time spent in open arms.

#### Studies with acute nicotine

**Fig 1:**
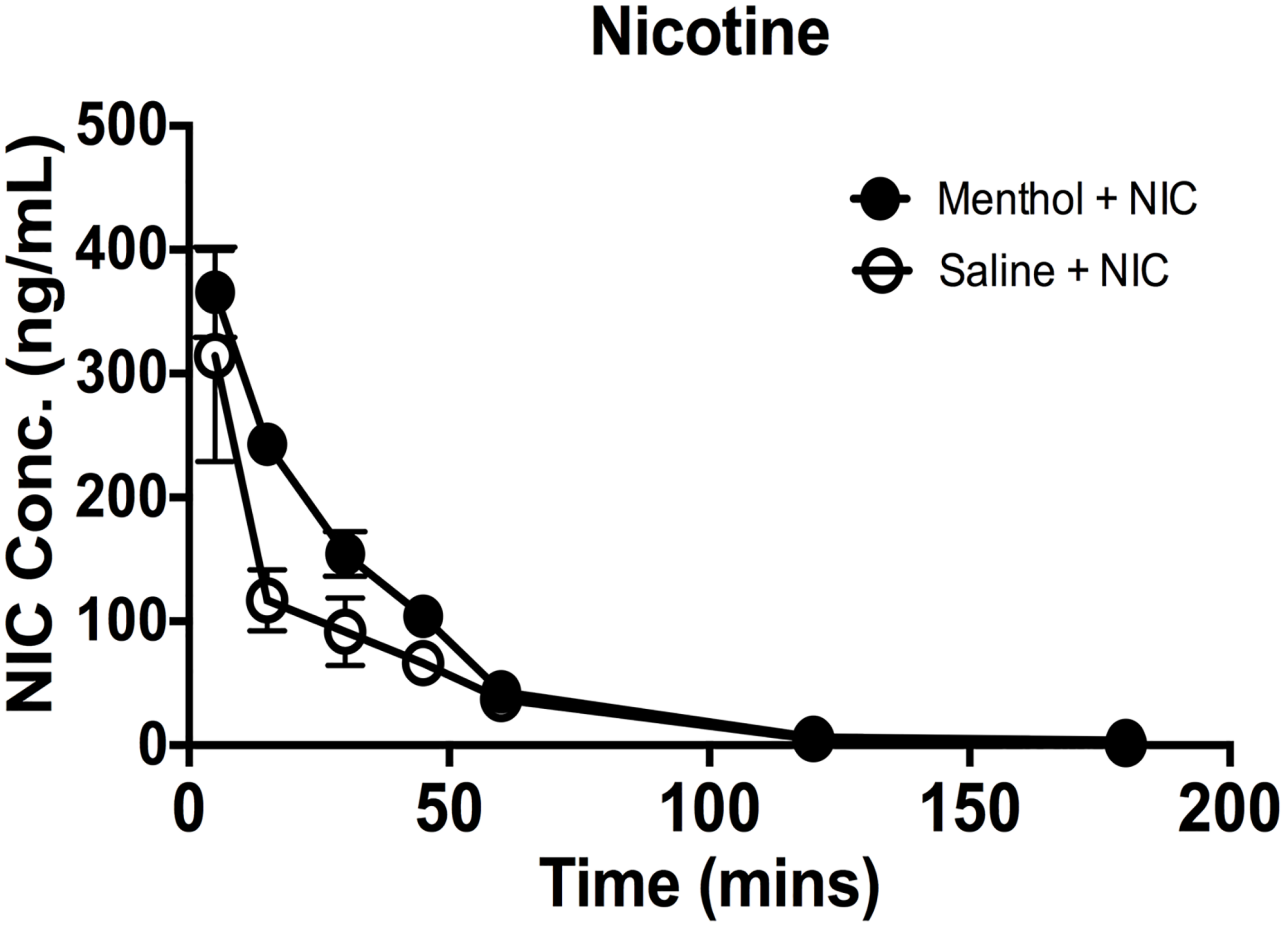
Time course of nicotine plasma concentrations in mice pretreated with menthol. Nicotine was administered (2.5 mg/kg s.c.), 30 min after pretreatment with vehicle or menthol (100 mg/kg i.p.). Each time point represents the mean ± SEM of 7 to 10 animals. For the vehicle pretreatment, values for nicotine plasma levels at 2 h were below the limits of detection. Each point represents the mean ± SE of 8–12 mice. Nic = nicotine.

We first determined the time-course and dose-dependency for menthol’s effects on nicotine-induced antinociception in the tail-flick test. Mice (n = 6–8) pretreated with vehicle or menthol (100 mg/kg, i.p.) and various time points (5, 30, 60 and 120 min) later, they were given nicotine (2.5 mg/kg, s.c.). We then evaluated the dose-dependency of menthol’s effects on nicotine-induced antinociception 45 min after nicotine administration in the tail-flick test. Mice (n = 6–8) pretreated with vehicle or different doses of menthol (10, 50, 100 or 200 mg/kg, i.p.) and 30 min later, they were given nicotine (2.5 mg/kg, s.c.). Mice were then evaluated for antinociception 45 min after nicotine administration (Fig 1A).

Nicotine time-course evaluation was conducted in mice (n = 6–8) pretreated with vehicle or menthol (100 mg/kg, i.p.) and 30 min later they were given nicotine (2.5 mg/kg, s.c.). Mice were then evaluated for antinociception [tail-flick and hot-plate tests: at 5, 15, 30, 45, 60, 120 and 180 min after nicotine administration] and for hypothermia (30, 45, 60, 75, 120, and 180 min after nicotine administration). Finally, to determine whether the methanol-enhanced nicotine effects were mediated by nicotinic receptors, animals were pretreated with either saline or mecamylamine (2 mg/kg, s.c.) followed by menthol (100 mg/kg, i.p.). Thirty min later, mice received nicotine at a dose of 2.5 mg/kg (s.c.) and were then tested at 45 min after injection.

#### Studies of nicotine withdrawal

The effect of menthol on nicotine withdrawal was tested using a modified precipitated nicotine withdrawal model as previously described [13, 14]. Mice were anesthetized with sodium pentobarbital (35 mg/kg i.p.) and implanted with Alzet osmotic mini pumps (Durect Corporation, Cupertino, CA) filled with (-)-nicotine or saline solution. The concentration of nicotine was adjusted according to animal weight and mini pump flow rate. Mice chronically infused with saline or nicotine (12 mg/kg/day) for 7 days and treated with vehicle or menthol (100 mg/kg, i.p. once a day). On day 8, 16–18 hr after mini pump removal, these mice were observed for physical and affective nicotine withdrawal signs as previously described [14]. Mice were first evaluated for 5 min in the plus maze test for anxiety-related behavior. The plus maze assessment was immediately followed by a 20 min observation of somatic signs. Next hyperalgesia was evaluated using the hot plate test. Mice were placed into a 10-cm wide glass cylinder on a hot plate (Thermojust Apparatus, Richmond, VA) maintained at 52°C. The latency to reaction time (jumping or paw licking) was recorded. The specific testing sequence was chosen based on our prior studies showing that this order of testing reduced within-group variability and produced the most consistent result [14]. All testing was conducted blind to group assignment.

### Brain protein extraction and Western blot analysis

The effect of menthol on nicotine-induced upregulation of nAChRs in different brain regions was performed after chronic administration of nicotine and menthol in 6 mice per condition. Mice were anesthetized with sodium pentobarbital (35 mg/kg i.p.) and implanted with Alzet osmotic mini pumps (Durect Corporation, Cupertino, CA) filled with (-)-nicotine or saline solution as described in Jackson et al. [14]. The concentration of nicotine was adjusted according to animal weight and mini pump flow rate. Mice chronically infused with saline or nicotine (12 mg/kg/day minipump, MP) for 7 days and treated with vehicle or menthol (100 mg/kg, i.p. once a day). Four groups were tested: saline (MP) + vehicle; saline (MP) + menthol (100 mg/kg); nicotine (12 mg/kg/day MP) + vehicle and nicotine (12 mg/kg/day MP) + menthol (100 mg/kg). Brains were isolated as described previously with minor modifications [15]. In brief, mice were anesthetized using 5% isofluorane followed by decapitation. Prefrontal cortex (PFC), striatum, and hippocampus were removed and dissected in an ice-cold dissection buffer (HBSS supplemented with 10 mM HEPES). To minimize intra-group variation during protein detection, common regions from two animals were pooled in triplicate for each experimental condition.

Membrane protein fractions were processed by brain tissue into 10 volumes of a cold homogenization buffer (0.32 M sucrose, 10 mM HEPES, 2 mM EDTA, pH 7.4 supplemented with a protease and phosphatase inhibitor cocktail [Roche]). The tissue was homogenized using a glass dounce homogenizer on ice until visibly dissociated (approximately 12–15 strokes). The homogenate was then centrifuged for 15 min at 1000 x g at 4°C and the supernatant fraction collected. A crude membrane protein fraction was isolated by spinning the supernatant fraction for 45 min at 200,000 x g at 4°C. The pellet was suspended in a solubilization buffer containing 50 mM HEPES, 2 mM EDTA, pH 7.4, supplemented with the protease phosphatase inhibitor cocktail (Roche) and gently mixed at 4°C for 1 hour.

Western blot detection of nAChRs from the membrane fraction was conducted on a nitrocellulose membrane using the following primary antibodies and dilutions: polyclonal anti-**β**2 (Santa Cruz) [0.2 μg/μL], polyclonal anti-**α**4 (Santa Cruz) [5 μg/μL], and rabbit polyclonal anti-GAPDH (Cell Signaling) [1 μg/μL] [15]. Species-specific peroxidase conjugated secondary antibodies were purchased from (Jackson-Immunoresearch). Protein bands were detected using SuperSignal West Pico Chemiluminescent Substrate (Thermo Scientific). SeeBlue (Life Technologies) was used as the molecular weight marker. Blots were imaged using the Gel Doc Imaging system (Bio-Rad). Band density analysis was performed using ImageJ version 10.2 (NIH). GAPDH was used as a loading control and a normalizing factor for protein loading across lanes. All samples were run in triplicates to obtain group averages.

### Plasma nicotine levels

To determine plasma nicotine levels in the different studies, blood samples were drawn by cardiac puncture. For the acute time-course studies, blood was drawn from mice at 5, 15, 30, 45, 60, 120, and 180 min after nicotine administration (2.5 mg/kg, s.c.). These mice were pretreated with either i.p. vehicle or menthol 30 min before nicotine administration.

Nicotine levels in adult male ICR mouse were also determined after pre-treatment with vehicle or menthol in the withdrawal study. Mice received nicotine (2 mg/kg, s.c.), three times daily for 4 days. On day 5, mice received one last nicotine dose, 1 hour later they were sacrificed, and blood samples were drawn.

Finally, blood was collected from the study of nAChRs protein levels after chronic minipump infusion of nicotine of nicotine (12 mg/kg/day) for 7 days. On day 8, mice were sacrificed, and blood samples were drawn.

Immediately afterwards the plasma samples were prepared by centrifugation at 3000 x g for 10 min and frozen at –20°C until analysis. To measure total nicotine levels (free and glucuronides) the samples were incubated with β-glucuronidase at a final concentration of 5 mg/ml in 0.2 M acetate buffer, pH 5.0, at 37°C overnight. After incubation the samples were processed and analyzed for nicotine levels by LC/MS/MS as described below.

### Nicotine LC/MS/MS analysis

#### Specimen extraction

To a 200 μl aliquot of plasma, 50 μl of internal standard containing 50 ng of nicotine-d4 in methanol was added with mixing. Then 100 μl of 5 M ammonium hydroxide was added to each sample followed by 2 ml methylene chloride. The samples were mixed for 2 minutes and then centrifuged for 5 min at 3000 rpm at a temperature of 4°C. The organic layer was transferred to a clean test tube. The aqueous phase was extracted twice more with 2 ml of methylene chloride. The organic phases were combined and 500 μl of 25 mM HCl in methanol was added. Samples then were evaporated to dryness under a gentle stream of nitrogen. They were reconstituted with 100 μl of mobile phase and placed in auto-sample (LC/MS/MS) vials for analysis.

#### Instrumental analysis

The LC/MS/MS system used was an Applied Bio systems 3200 Qtrap with a turbo V source for TurbolonSpray with a Shimadzu SCL HPLC system controlled by Analyst 1.4.2 software. The chromatographic separation was performed using an Hypersil Gold, 3 mm X 50 mm, 5 micron (Thermo Scientific, USA). The mobile phase contained 10 mM ammonium formate; methanol (10:90 V/V) and was delivered at a flow rate of 0.5 ml/min. The acquisition mode used was multiple reaction monitoring (MRM) in a positive mode. Transition ions monitored for nicotine (163>130; 163>117) and nicotine-d4 (167>134). The total chromatographic separation time for each extract injection was 2 min. A calibration curve ranging from 12.5 ng/ml to 500 ng/ml was constructed for each compound based on linear regression using the peak area ratios of the drug to its deuterated internal standard.

### Statistical analysis

Assessment of *in vivo* nicotine levels for the entire time-course was not possible from individual animals due to limited blood volume; therefore each time point represented data from multiple individual mice. The *in vivo* pharmacokinetic parameters were determined using non-compartmental analysis: AUC_0-3hr_ was determined by the trapezoidal rule up to the last measured concentration: C_3hr_, while C_max_ was determined as the maximum (of the mean) concentration. Elimination half-life (t_1/2_) was estimated using a nonlinear mixed effects model to account for absorption using the ‘NLME’ package of ‘R’ (single dose, first order absorption, linear elimination). The pharmacokinetic parameters and statistical analyses were estimated by randomization testing using SAS version 8.2. Statistical analysis of all behavioral studies was performed using analysis of variance (ANOVA) followed by Tukey’s post hoc test or t-test when appropriate. Behavioral time-course studies with menthol and nicotine were analyzed by a one-way or two-way ANOVA with repeated measure. Dose-response studies with menthol and nicotine were analyzed by a one-way ANOVA as well as the withdrawal studies. All differences were considered significant if at p < 0.05. The GraphPad Prism program was used for graphical representations and statistical analysis (GraphPad Software Inc., San Diego CA).

## Results

### Effects of menthol on nicotine plasma level after acute *in vivo* treatment

We tested the effect of menthol on in vivo plasma nicotine levels after administration of nicotine (2.5 mg/kg s.c.) in mice pretreated with vehicle or menthol (100 mg/kg, i.p.). Intraperitoneal injection of menthol had no effect on the nicotine’s C_max_, or the plasma nicotine half-life; however, it significantly increased its AUC (181 ± 7) compared with vehicle treatment (119 ± 8) and reduced its clearance (Fig 1 and Table 1).

**Table 1:** Impact of menthol treatment on nicotine pharmacokinetic parameters. Pharmacokinetic parameters of plasma nicotine in mice pre-treated i.p. with menthol (100 mg/kg) or vehicle 30 minutes prior to nicotine (2.5 mg/kg, s.c.). Results were derived using data (Fig 1 [time-concentration graph]).

	Nicotine (mean ± SEM)	
	Saline	Menthol
**AUC**_**0-3hr**_ **(ng • hr/ml)**	119±8*	181±7
**C**_**max**_ **(ng/ml)**	314±85	366±36
**t**_**1/2**_ **(min)**	16.9±4.6	23.9±4.6
**Cl/f (L/min)**	11.2±0.75*	6.9±0.27

*p < 0.05 compared to Menthol.

### Effects of menthol on nicotine acute pharmacological effects (antinociception and hypothermia): Time-Course, Potency and Blockade Studies

Menthol (100 mg/kg) given i.p. was evaluated for its ability to enhance nicotine-induced antinociception (tail-flick and hot-plate procedures) and hypothermia. We first examined the impact of menthol on the time course of nicotine pharmacological effects.

**Fig 2:**
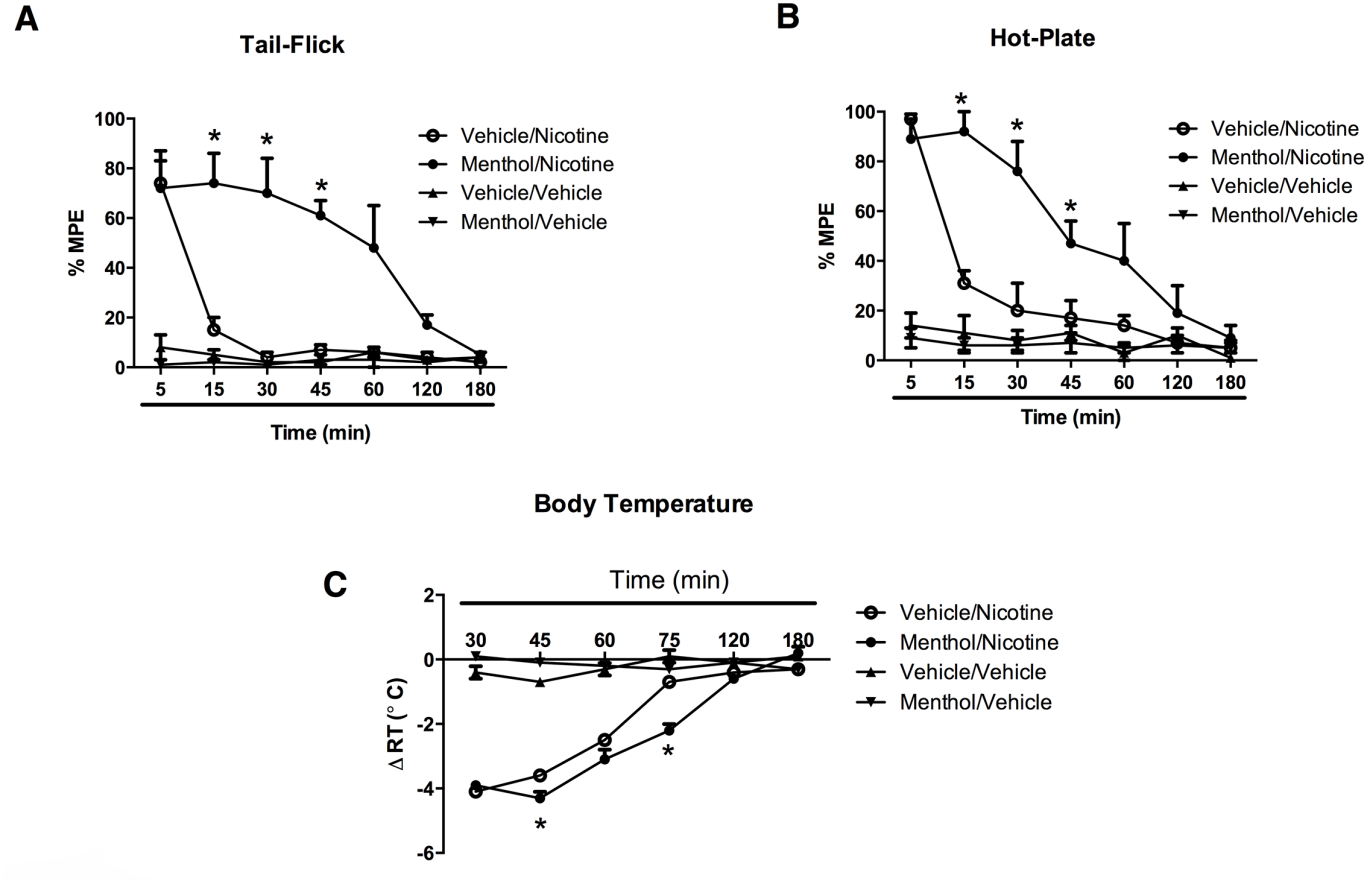
Effects of menthol on the time course of nicotine’s effects in (A) the tail-flick test, (B) the hot-plate assay, and (C) body temperature in mice. Animals were pretreated with either menthol (100 mg/kg i.p.) or vehicle and 30 min later received nicotine (2.5 mg/kg, s.c.). A control group (vehicle/vehicle) is also represented in all three tests. Mice were tested at different time points after injection. Each point represents the mean ± SE of 8–12 mice. *p < 0.05 compared with vehicle/nicotine group.

**Fig 3:**
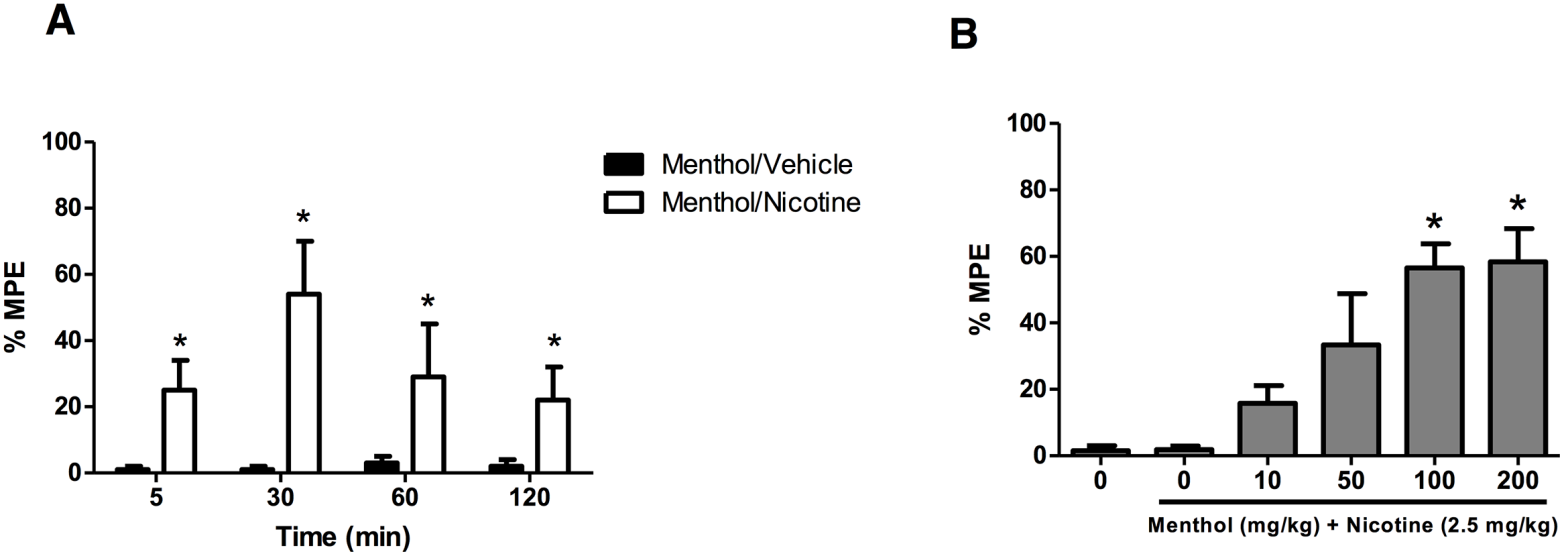
Effect of (A) pretreatment time and (B) dose of menthol’s effect in the tail-flick. **(A)** Mice pretreated with vehicle or menthol (100 mg/kg, i.p.) and various time points (5, 30, 60 and 120 min) later, they were given nicotine (2.5 mg/kg, s.c.). Mice were then evaluated for antinociception 45 min after nicotine administration. **(B)** Mice pretreated with vehicle or different doses of menthol (10, 50, 100 or 200 mg/kg, i.p.) and 30 min later, they were given nicotine (2.5 mg/kg, s.c.). Mice were then evaluated for antinociception 45 min after nicotine administration. Each point represents the mean ± SE of 8–12 mice. *p < 0.05 compared with control.

Mice were given menthol and 30 min later they received nicotine and were then tested at different times for antinociception and hypothermic responses. At this dose nicotine (2.5 mg/kg, s.c.) alone induced a relatively short-lived antinociceptive effect in the tail-flick (30 min- Fig 2A) and hot-plate (45 min- Fig 2B) tests. Additionally, a decrease in body temperature was observed after nicotine s.c. administration that lasted for 75 min (Fig 3C). When mice were pretreated with menthol (100 mg/kg, i.p.), the effects of nicotine were significantly prolonged in both analgesic tests; in tail-flick [F(2, 12) = 10.79, p = 0.0021] and hot-plate [F(2, 12) = 8.750, p = 0.0051] tests, nicotine-induced antinociception did not disappear completely till 120 and 180 min in the tail-flick and hot-plate tests, respectively (Fig 2A and 2B). On the other hand, the effects of nicotine on body temperature were still significant until the 120 min time point (Fig 2C) [F(2, 10) = 9.064, p = 0.0169]. Interestingly, the plasma nicotine concentrations achieved during the first 45 min in the menthol-treated group (Fig 1) are comparable or even larger than the nicotine concentrations at 5–45 min of vehicle-treated group when mice are pretreated with menthol, whereas the efficacy differences in the three pharmacological measures between these groups are quite pronounced (Fig 2) in the tail-flick [t(3) = 4.260, p = 0.0237], hot-plate [t(3) = 5.422, p = 0.0123], and in body temperature test [t(3) = 3.873, p = 0.0305].

Based on the nicotine time-course results, subsequent studies examining the time-course and potency of menthol’s effects on nicotine. Mice were given menthol at different times and then received nicotine (2.5 mg/kg, s.c.). The mice were then tested 45 min after nicotine administration. As illustrated in Fig 3A, the potentiation of nicotine’s antinociceptive effects by menthol pretreatment in the tail-flick test was time-dependent with maximum enhancement occurring between 5 and 30 min, t(10) = 2.472, p = 0.0330 and t(10) = 3.355, p = 0.0073, respectively. A similar time-course was seen in the hot-plate and with nicotine-induced hypothermia. Based on the time-course results, subsequent studies were carried out by pretreating the mice with menthol 30 min before nicotine. We then determined menthol’s potency of enhancing nicotine’s effects at this pretreatment time. As showed in Fig 3B, menthol dose-dependently enhanced nicotine-induced antinociception in the tail-flick [F(3, 23) = 6.954, p = 0.0022]. By itself, menthol did not significantly change tail-flick or hot-plate basal latencies, or body temperature at the indicated doses and times (Fig 2). Furthermore, the antinociceptive response in tail-flick [t(10) = 5.923, p = 0.0001] and hot-plate test [t(10) = 3.505, p = 0.0057], and hypothermic effects [t(10) = 4.118, p = 0.0021], of the menthol/nicotine combination were totally blocked by a pretreatment with mecamylamine (2 mg/kg), a non-competitive nicotinic antagonist (Table 2).

**Table 2:** Blockade of menthol’s effects on nicotine acute pharmacological responses after administration in mice. Animals were pretreated with either saline or mecamylamine (2 mg/kg, s.c.) followed by menthol (100 mg/kg, i.p.). Thirty min later, mice received nicotine at a dose of 2.5 mg/kg (s.c.) and were then tested 45 min after injection. Menthol = menthol at 100 mg/kg, i.p.; Mec: Mecamylamine at 2 mg/kg, s.c.; Nic: Nicotine at 2.5 mg/kg, s.c.; Sal: Saline; Veh: vehicle. Each point represents the mean ± SE of 8 to 10 mice

Treatment	Tail-flick Test	Hot-plate Test	Hypothermia (Δ°C)
(mg/kg)	% MPE ± SEM	% MPE ± SEM	(Mean ± SEM)
**Sal/Veh/Sal**	3±1	6±3	-0.1±0.1
**Sal/menthol/Sal**	0±0	9±4	-0.1±0.1
**Mec/menthol/Sal**	4±1	6±3	-0.2±.01
**Mec/Veh /Sal**	6±2	5±3	-0.4±0.1
**Mec/Veh /Nic**	5±2	7±3	-0.2±0.2
**Sal/Veh/Nic**	6±4	15±7	-3.1±0.4* #
**Sal/menthol/Nic**	59±9* #	52±12* #	-3.1±0.3* #
**Mec/menthol/Nic**	3±2	7±4	-0.8±0.5

*p <0.05 compared to Saline/Vehicle/Saline.

^#^p <0.05 compared to Saline/menthol/Saline.

**Fig 4:**
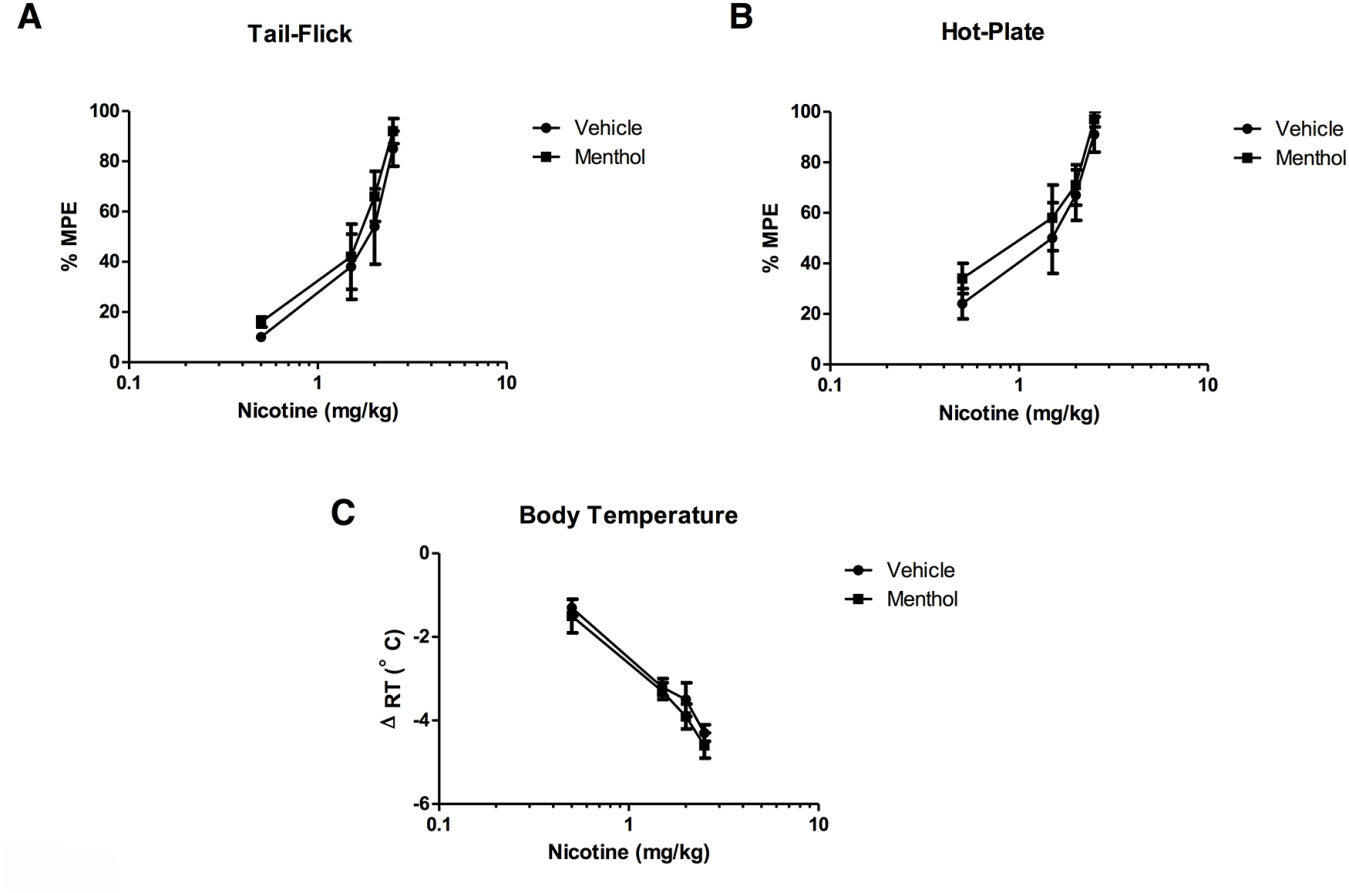
Effects of menthol pretreatment on nicotine-induced antinociception and hypothermia dose-response curves in mice. Vehicle or menthol (100 mg/kg, ip) was administered 30 min before various doses of nicotine (0.5, 1.5, 2, and 2.5 mg/kg s.c.) and mice were tested in **(A)** the tail-flick test, **(B)** the hot-plate test, and **(C)** hypothermia. Each point represents the mean ± SE of 8–12 mice.

Finally, we evaluated the effects of menthol on nicotine pharmacological potency at peak-time effect. Mice pretreated with menthol (100 mg/kg, ip) were challenged 30 min later with different doses of nicotine (s.c.) and evaluated for antinociception in the tail-flick and hot-plate tests (5 min after nicotine injection) and hypothermia (20 min after nicotine injection). As seen in Fig 4, menthol pretreatment did not significantly shift nicotine-induced antinociception (tail-flick and hot-plate tests) and hypothermia dose-response curves. Similarly, nicotine’s potency (ED_50_ value) was not significantly modified by menthol pretreatment (Table 3).

**Table 3:** Summary of the potency of nicotine in male and female mice in different behavioral tests. Animals were pretreated with either vehicle or menthol (100 mg/kg, i.p.) followed by nicotine (2.5 mg/kg s.c.) at different doses and then tested 5 min (tail-flick and hot-plate tests) or 20 min later (hypothermia). Each point represents the mean ± SE of 6 to 8 mice.

Effect	Nicotine (ED_50_ mg/kg)	Menthol+Nicotine (ED_50_ mg/kg)
**Tail-flick**	1.7 (1.2–2.3)	1.3 (1.0–1.7)
**Hot-plate**	1.1 (0.8–1.5)	0.9 (0.7–1.2)
**Hypothermia**	1.3 (1–1.7)	1.1 (0.9–1.4)

ED_50_ values (±CL) were calculated from the dose-response curve of the different groups and expressed as mg/kg.

### Effects of menthol on precipitated nicotine withdrawal signs in mice

In this part of the study and based on our previous results, we examined whether greater nicotine exposure by menthol treatment would enhance nicotine withdrawal, an important measure of nicotine dependence. Mice were tested for somatic withdrawal responses, anxiogenic-like effects in the elevated plus maze, and hyperalgesia in the hot plate test.

**Fig 5:**
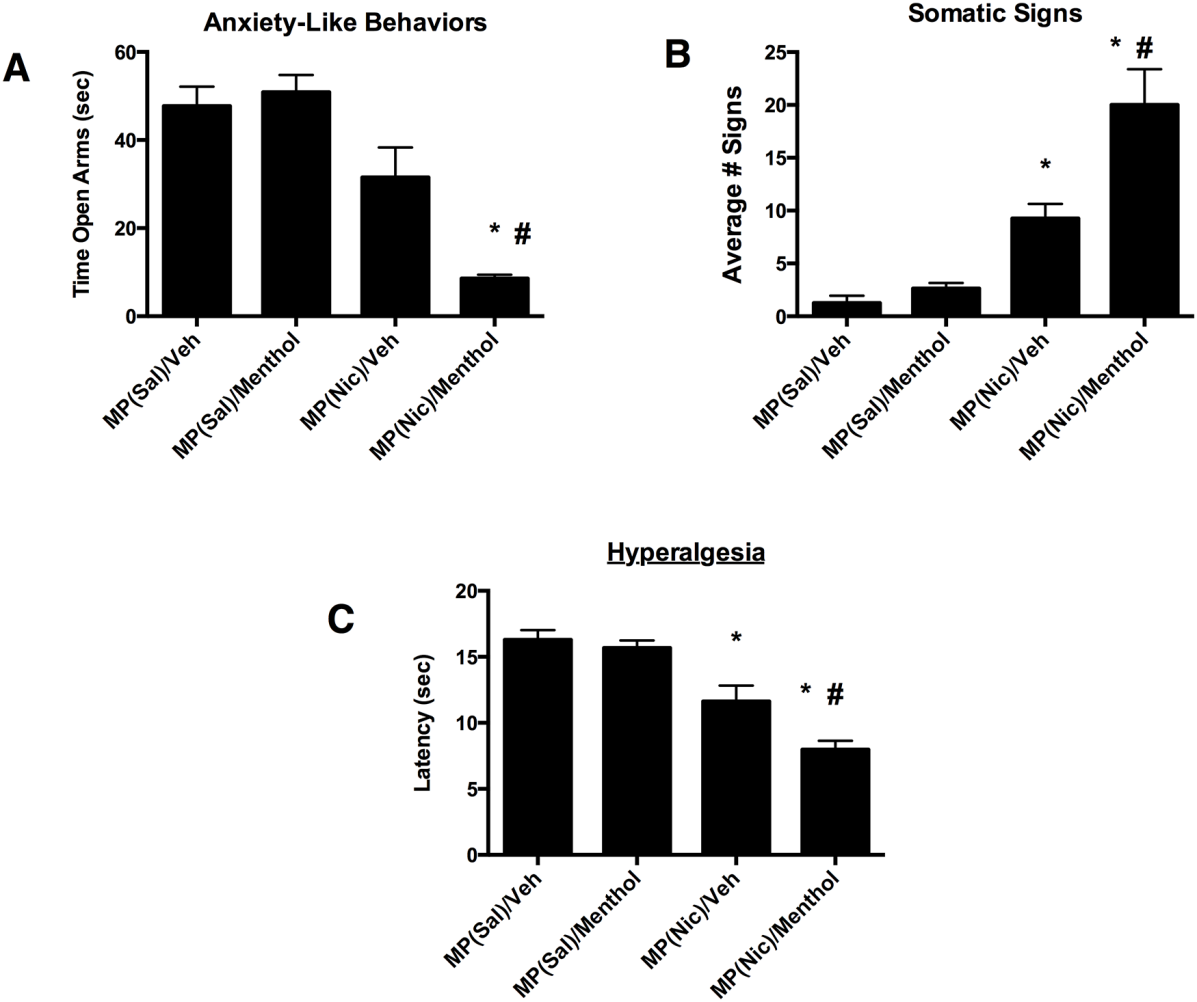
Effects of menthol on nicotine withdrawal signs. (A) Elevated plus-maze test, (B) Somatic signs and in (C) Hyperalgesia. Withdrawal from nicotine induced a: A) no significant change in anxiety- related behavior, B) significant increase in somatic signs, and C) a significant decrease in hot plate latency. Compared to vehicle, pretreatment with menthol (100 mg/kg, i.p. for 7 days) A) significantly increased expression of anxiety-related behavior; B) a further increase in somatic signs, and C) a decrease in hot plate latency in mice. Each point represents the mean ± S.E.M. of 6–8 mice per group. *P < 0.05 compared with control MP/Saline/Vehicle group. #*P* < 0.05 compared with MP/Nicotine/ Vehicle group. MP = minipump; Nic = nicotine; Sal = saline; Veh = vehicle.

Physical (somatic signs and hyperalgesia) and affective (anxiety-related behavior) signs were measured in mice following 18–24 h withdrawal from chronic nicotine and treatment with either menthol or vehicle. Results are shown in Fig 5. In mice exposed to vehicle, nicotine withdrawal did not significantly increase anxiety-related behavior in the plus maze [F (3, 26) = 7.26; P < 0.625] (Fig 5A), but did increase expression of somatic withdrawal signs [F (3, 28) = 21.45; P < 0.0001] (Fig 5B), and decreased response latencies in the hot-plate test [F (3, 26) = 20.65; P < 0.0001] (Fig 5C). The animals pretreated with menthol exhibited a significant decrease in the average time spent in open arms [F(3, 20) = 54.95, p<0.0001] (Fig 5A), and a further increase in somatic withdrawal signs [F(3, 20) = 167.3, p<0.0001] Fig 5B). Additionally, menthol pretreatment caused significant increased hyperalgesic effects compared to vehicle pretreatment [Fig 5C; F(3, 20) = 32.72, p<0.0001]. The increase in nicotine withdrawal intensity in the group pretreated with menthol was accompanied by an increase in nicotine plasma levels (Table 4). In summary, menthol pretreatment of nicotine-treated mice resulted in enhanced withdrawal intensity of all signs.

**Table 4:** Nicotine plasma levels in adult male ICR mouse after pre-treatment with vehicle or menthol in the withdrawal testing. Mice received nicotine (12 mg/kg/day) for 7 days. On day 8, mice were sacrificed and blood samples were drawn. Values are shown as mean ± S.E.M. (n = 6-8/group)

Group	Nicotine (ng/ml)
**Vehicle/Nicotine**	56 ± 19
**Menthol/Nicotine**	143 ± 31*

*P<0.05 compared to vehicle/nicotine group.

### Effects of menthol on nicotinic receptors protein levels after chronic exposure in mice

**Fig 6:**
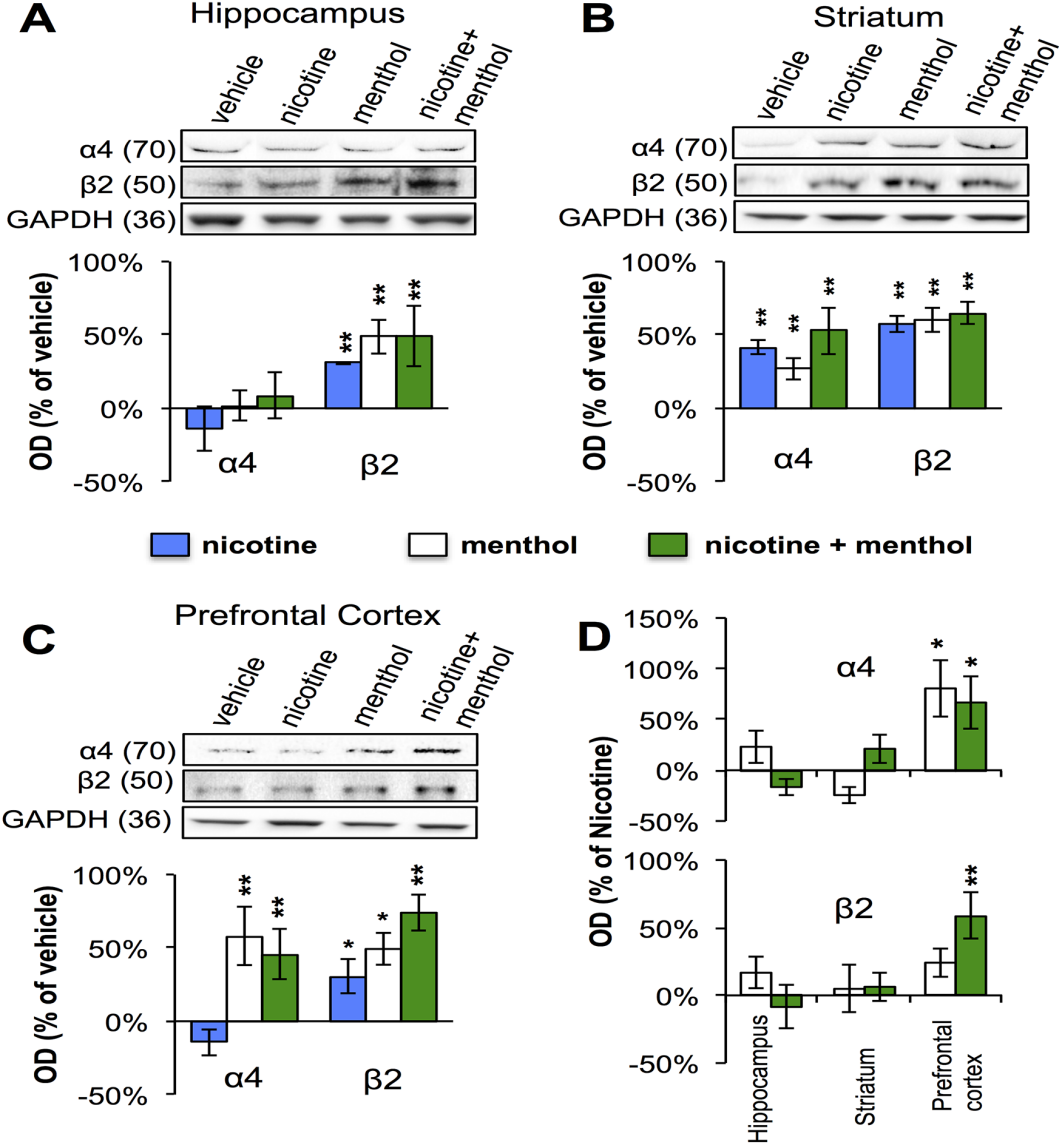
Menthol mediated changes in nAChR expression in the brain. Western Blot detection of **α**4 and **β**2 nAChR subunits in membrane fractions of treated mice. Representative immunoblots showing the expression of **α**4 and **β**2 nAChR subunit bands in **(A)** Hippocampus, **(B)** Striatum, **(C)** Prefrontal Cortex, and **(D)** all three regions when expressed relative to nicotine treatment alone. GAPDH was used as a loading control and a normalizing factor for protein loading across lanes. Histograms show average values of optical density (OD) measurements of the immunoreactive bands relative to the vehicle lane (n = 6 mice per group; and all samples were run in triplicates). Student’s test *p < 0.05, **p < 0.01.

Since chronic nicotine treatment elicits a brain region-selective increase (up-regulation) in the number of **α**4**β**2 nicotinic receptors (for review, see Govind et al., [16]), we examined whether menthol exposure would modulate nicotine-induced up-regulation of **α**4 and **β**2 nAChR subunit proteins in key brain regions of addiction. As shown in Fig 6, mice chronically infused with saline or nicotine (12 mg/kg/day) were treated with i.p. vehicle or menthol (100 mg/kg, once a day) for 7 days and three brain regions were removed. Based on optical density analysis of reactive bands on a western blot, statistically significant increases were seen in the expression of **β**2 in the hippocampus in response to nicotine, menthol, and both nicotine and menthol. No change in the expression of **α**4 was seen in the same hippocampal tissue. In the striatum on the other hand, an increase in **α**4 and **β**2 levels was detected across all treatment groups with a statistically significant rise in the level of **α**4 expression in mice treated with menthol or nicotine (Fig 6B). Last, the levels of **α**4 and **β**2 proteins were also significantly elevated in cortical tissue under menthol and nicotine treatments with no detectable change in **α**4 expression in nicotine treatment alone (Fig 6C). The findings demonstrate an effect of nicotine both alone and in combination with menthol on the expression of these nAChRs in various brain regions. When normalized to the vehicle treatment conditions a differential effect of nicotine and menthol on nAChR expression is revealed in the adult rodent brain. Menthol treatment in particular appears to significantly enhance the expression of the two nAChR subunits in the prefrontal cortex relative to nicotine treatment alone (Fig 6D).

## Discussion

Our study with menthol in mice confirms previous results and provides several novel findings. First, we have confirmed that menthol induced a significant increase in nicotine systematic exposure in mouse. Second, we show that the increase in nicotine levels has functional consequences and leads to a prolongation of nicotine acute pharmacological effects and an enhancement of nicotine withdrawal intensity. Finally, we show that chronic menthol treatment differentially regulates **α**4 and **β**2 nAChR levels in various brain regions.

Our finding that menthol exposure in mice increased nicotine plasma AUC and reduced its total clearance confirms previous results reported in smokers. Indeed, similar changes in nicotine AUC and total clearance were reported between the mentholated and the nonmentholated cigarette smoking groups [1]. After exposure to menthol, the increase in nicotine plasma AUC in mice was significant but rather modest (1.5 fold) and the treatment did not significantly prolong the nicotine plasma levels (Table 1). At time point 60 min and beyond, the plasma levels for the two treatment groups (nicotine and nicotine-menthol) totally overlapped. Overall, these results in mice are consistent with reports showing that menthol is a weak inhibitor of CYP2A6 enzyme in rats and human microsomes [17], [18]. Menthol is also rapidly glucuonidated which might alter the metabolism of nicotine via competing with the minor nicotine-glucuronide pathway; while smoking menthol cigarettes there were lower ratios of the nicotine-glucuronide/nicotine than in the non-menthol condition [19]. In contrast to the modest pharmacokinetic impact of menthol in vivo, there were dramatic enhancements in the pharmacological effects of nicotine (body temperature and analgesia) after menthol treatment. The effects of menthol on nicotine were dose-dependent and reached maximal enhancement at a dose of 100 mg/kg. Furthermore, the duration of menthol-induced potentiation of nicotine’s antinociceptive and hypothermic lasted close to 180 min after drug pretreatment. However, the pharmacological potency of nicotine did not differ between menthol-treated and non-menthol treated mice. While changes in nicotine levels and clearance may explain partly the enhancement of pharmacological effects, the dissimilarity between nicotine plasma time-course and behavioral time-course observed suggests the involvement of other pharmacological mechanisms. It is also possible that brain nicotine levels, not measured in our study, contributed to a greater extent to this phenomenon.

In our study we also evaluated the impact of menthol exposure on drug withdrawal, an important aspect of nicotine dependence. Chronic nicotine administration in rodents induces the appearance of physical (somatic signs and hyperalgesia) and affective (anxiety-related behavior) nicotine withdrawal signs. Administration of menthol to nicotine-exposed mice significantly enhanced the intensity of all three nicotine withdrawal signs measured in our study: somatic signs, anxiety-related behaviors and hyperalgesia. This is the first study that reports on that systemic menthol modulates nicotine dependence in rodent models. Multiple mechanisms could underlie the interaction between menthol and nicotine’s behavioral actions. First, the potentiation of withdrawal intensity by menthol was consistent with the accompanying significant increase in nicotine plasma levels in these mice. This is in line with the increase in nicotine withdrawal after pretreatment of mice with a CYP2A5 inhibitor [20]. Second, the change in nicotine withdrawal could also be due to the ability of menthol to block nAChRs. Indeed, studies have shown that menthol attenuates nicotinic responses at **α**4**β**2 and **α**7 nAChR subtypes in a non-competitive manner [9], [21]. The dose of menthol used in our studies yielded brain levels that are consistent with such mechanism as recently reported in mice [22]. Finally, the regulation of brain α **α**4**β**2 nAChRs levels by menthol after chronic treatment may have also played a role. Our results showed for the first time that chronic in vivo treatment with menthol induced an upregulation of **α**4**β**2 nAChRs in specific brain regions. It is possible that menthol affects other nAChRs including the **α**7 nAChR in the brain presenting an important future direction of research. Interestingly, temporal correlation between **α**4**β**2 nAChRs upregulation and nicotine withdrawal in rodents and humans were reported [23], [24].

Our results showing that menthol induces upregulation of **β**2 containing nAChRs in the hippocampus, striatum, and prefrontal cortex are consistent with a recent study that showed that menthol smokers have greater up-regulation of nAChRs than non-menthol smokers in similar brain regions [12]. However, the study of Brody et al. did not include denicotinized mentholated cigarettes as a control. Interestingly, in contrast to **β**2 subunits, the changes in the expression of **α**4 subunits were not seen in all brain regions suggesting that nicotine and/or menthol induced upregulation of these nAChRs is cell autonomous. In this study we present new evidence on an effect of menthol on nAChR levels in rodents. This effect is consistent with the notion that menthol operates as a non-competitive nAChR blocker [21], thereby impacting receptor expression and function. Interestingly, menthol enhanced nicotine-induced upregulation of **α**4**β**2 nAChRs in the prefrontal cortex only, suggests a role for chronic menthol in cortical aspects of nicotine addiction and withdrawal. In fact our data suggests that menthol treatment upregulation of nAChRs in the prefrontal cortex are independent of nicotine treatment.

In summary, menthol treatment leads to a modest increase in nicotine’s bioavailability, a substantial increase in nicotine pharmacological time-course and nicotine withdrawal intensity as well as an up-regulation of α4β2* nAChRs in mice, consistent with menthol cigarette smoking leading to greater up-regulation of these receptors than non-menthol cigarette smoking. These findings may help explain the relative severity of dependence on menthol cigarettes.
